# Strengthening the Role of Genomics in Global Health

**DOI:** 10.1371/journal.pmed.0010040

**Published:** 2004-12-28

**Authors:** Tara Acharya, Abdallah S Daar, Halla Thorsteinsdóttir, Elizabeth Dowdeswell, Peter A Singer

## Abstract

How genomics and related health biotechnologies can improve the health of the poor and contribute towards meeting the Millenium Development Goals

Development experts and policy makers agree that investment in science and technology is important for economic growth and development. The 2001 United Nations (UN) Development Programme report, *Making New Technologies Work for Human Development*, identified technical progress as the largest factor in reducing mortality rates and improving life expectancy from 1960 to 1990 [1]. A May 2004 report to UN Secretary-General Kofi Annan from the InterAcademy Council on Science and Technology Capacity supports the view that mobilization of sound scientific knowledge and evidence-based principles is needed to address critical world issues such as poverty, disease, and the effects of globalization and economic transformation [2]. Annan himself has drawn attention to the importance of science capacity for global development, observing that “no nation can afford to be without its own [science and technology] capacity” [3].

This capacity is essential if the world is to achieve the UN Millennium Development Goals (MDGs), which were adopted by all UN members in 2000 in a commitment to promote sustainable development and eliminate poverty in the world. As part of the Millennium Project, the UN established task forces to come up with strategies to help developing countries achieve the MDGs. One of these is the Task Force on Science, Technology, and Innovation (Task Force 10), created in recognition of the fact that many of the goals, especially those related to health and the environment, cannot be realized without a strong contribution from science and technology [4]. In a report titled *Genomics and Global Health*, presented recently to Task Force 10, we addressed how genomics and related health biotechnologies can improve global health and contribute towards meeting the MDGs [5]. The report shows how the world can unite in a global approach to meet these objectives and what steps developing countries themselves are taking to harness these technologies. The main findings of the report are summarized in our conclusions below.

## Genomics Can Contribute to the MDGs

Genomics refers to the powerful new wave of health-related life sciences energized by the human genome project and the knowledge and tools it is spawning. It is a relatively new science that has tremendous potential to address health problems in developing countries.

The role of genomics in global health has been highlighted previously in the World Health Organization's 2002 report [6], and explored further in a technology foresight exercise by the University of Toronto Joint Centre for Bioethics [7]. Genomics-related technologies, including DNA sequencing and bioinformatics, were once considered expensive, exotic, and applicable only to wealthy nations, but this perception has been changing over the past few years. Through the efforts of companies and institutions worldwide, certain applications have become simpler and cheaper to the point that they can start replacing older technologies that are currently used for health care in poorer nations. Such simple and easy-to-use tests are being developed for tuberculosis, hepatitis C, HIV, malaria, and other diseases (e.g., the OptiMAL rapid malaria test [http://www.malariatest.com/]). Recombinant vaccines, a result of genetic engineering, promise to be safer, cheaper, and possibly easier to store and transport than traditional vaccines [7]. Microorganisms with remarkable biochemical properties show promise of being able to reduce pollution, making water safer to drink [8]. Table 1 provides a snapshot of how genomics and related biotechnologies can support some important MDGs [9]; a more complete discussion can be found in our report [5].

**Table 1:**
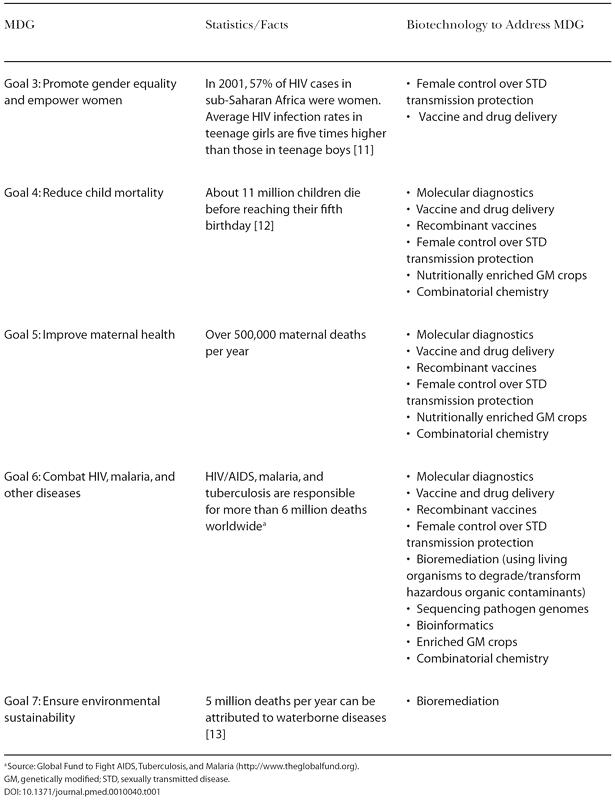
Genomics and Related Biotechnologies Can Support the MDGs

^a^Source: Global Fund to Fight AIDS, Tuberculosis, and Malaria (http://www.theglobalfund.org)

GM, genetically modified; STD, sexually transmitted disease

## What the International Community Can Do

In *Genomics and Global Health* [5], we argue that genomics knowledge should be considered a global public good [10]. We need to establish a governance mechanism that fosters a balance between genomics knowledge as a public good and the application of this knowledge to foster private-sector interests. We propose the creation of a global partnership, the Global Genomics Initiative (GGI), to promote genomics for health. We see this as a network of industry leaders, academics, concerned citizens, non-governmental organizations, and government officials, with strong representation from the developing world. The proposed GGI would highlight broad actions that should be taken at the global level to apply genomics to development issues in this new era of globalization. Participation in the GGI would strengthen capacity in biotechnology worldwide by increasing international and inter-sectoral exchange of knowhow, and encouraging partnerships between countries.

The GGI could also facilitate the sharing of good practices. For example, the Canadian Prime Minister, Paul Martin, in his February 2004 reply to the Speech from the Throne, set a long-term target for Canada to devote 5% of its research and development spending to the challenges of developing countries. If successfully implemented and replicated by other industrialized countries, this target could make a real difference to global health.

## What Developing Countries Can Do

**Figure 1:**
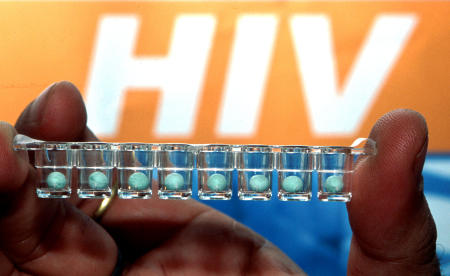
A Rapid Test for HIV Used in Jaipur, India Biotechnology has a vital role to play in developing better diagnostic tools for diseases such as HIV, tuberculosis, and malaria. (Photo: World Health Organization/P. Virot)

Our report concludes that the key actors are developing countries themselves. We explore how to put genomics and related technologies to work in developing countries within the next 5–10 years. Developing countries with the scientific capacity and institutional arrangements that allow creation, utilization, adaptation, and diffusion of genomics are well positioned to harness this new science for development (Figure 1). Learning is important for building genomics capacity, and is central to the creation of national systems of innovation (NSIs) in biotechnology in developing countries.

Today there are examples of strategies that some developing countries, including China, Cuba, Brazil, India, and South Africa, have followed to institute learning processes that are helping them to build NSIs in biotechnology. China seized the opportunity to take part in the Human Genome Project and quickly set up major institutions in genomics, such as the Beijing Genomics Institute, equipped with state-of-the-art sequencing facilities and computers. It has also followed a strategy of private-sector development in line with the NSI framework. Because of a government policy encouraging their return, Chinese expatriates are increasingly active in setting up small health biotechnology firms, bringing to the country knowledge from the world's major centers for genomics and related technologies.

The government in Cuba became interested in biotechnology in the early 1980s when the field was still in its infancy and created an interdisciplinary group, the Biological Front, to explore the possibilities of the technology for Cuba. It has continued to support biotechnology even during periods of economic hardship, set up institutions with research, development, and manufacturing facilities, and encouraged linkages between these institutions by setting up a biotechnology cluster, the West Havana Scientific Pole. Encouraging linkages has been a core policy of the government in Cuba, and its health biotechnology development has benefited from the ties with a strong public health system.

Brazil has a relatively long history of supporting science and technology, and the country is increasingly focusing on genomics and related technologies. The lack of commercialization of its cutting edge science and technology has been a weakness of the system in Brazil, but the country is now trying to overcome this weakness by proposing an “Innovation Law” that encourages cooperation between universities and the private sector.

Since its independence in 1947, India has followed a vision to improve the quality of life of its people by emphasizing science and technology. Limited resources and a patenting system that did not allow patenting of pharmaceutical products but only patenting of processes encouraged firms to come up with low-cost process innovation. This has resulted in health products such as the Shanvac-B hepatitis B vaccine, which is produced in India for a fraction of the cost in developed countries.

The South African government's Biotechnology Advisory Committee recognizes that successful commercialization of public-sector-supported research and development requires strong linkages within the NSI. The committee has recommended the creation of several Regional Innovation Centres to act as nuclei for the development of biotechnology platforms that can effectively launch new products and services. These strategies will provide important lessons for many other developing nations as they begin participating in the genomics revolution.

## Six Conclusions to Our Report

First, the development gap between developing countries and the industrialized world continues to grow. The international community is beginning to promote science and technology to reduce this gap. The genomics revolution holds tremendous potential to improve health in developing countries and, if harnessed appropriately, could help to reduce the development divide.

Second, genomics and related biotechnologies can help to achieve the UN MDGs. Fast, accurate molecular diagnostic devices, safer recombinant vaccines, female-controlled vaginal microbicides, and low-cost bioremediation tools are some examples of biotechnologies that can have an impact.

Third, genomics knowledge has the characteristics of a global public good. In order to harness the benefits of genomics for development, the developing world needs, above all, access to genomics knowledge. Fourth, the promotion of the science of genomics as a global public good and the encouragement of global knowledge flows could best be achieved through international partnerships. A GGI involving an international partnership of public and private entities from both developed and developing countries could catalyze genomics knowledge and learning worldwide.

Fifth, countries that have genomics capacity are best positioned to take advantage of the genomics revolution to meet their health needs. For the transfer of technologies to be effective and sustainable, it must be accompanied by transfer of science and knowledge. As well, receiving countries must have the capacity to absorb and use the technology.

And sixth, learning is important for building genomics capacity, and is central to the creation of NSIs in biotechnology in developing countries. These countries can strengthen the building blocks of the NSI framework by doing the following: re-energizing academic institutions and public-sector research to strengthen their science base; training people and building human capital to use, adapt, and innovate biotechnologies; encouraging regional and international cooperation to create new channels for knowledge exchange and trade; improving the policy environment (including intellectual property laws and regulation) to encourage the building of capacity; and fostering the growth of the private sector, encouraging it to address local health needs, and strengthening linkages between public and private sectors to create new biotechnology goods and services.

## Abbreviations

GGI: Global Genomics Initiative
MDG: Millennium Development Goal
NSI: national system of innovation
UN: United Nations
